# Species identity matters when interpreting trophic markers in aquatic food webs

**DOI:** 10.1371/journal.pone.0204767

**Published:** 2018-10-05

**Authors:** Zachary S. Feiner, Carolyn J. Foley, Harvey A. Bootsma, Sergiusz J. Czesny, John Janssen, Jacques Rinchard, Tomas O. Höök

**Affiliations:** 1 Department of Forestry and Natural Resources, Purdue University, West Lafayette, Indiana, United States of America; 2 Illinois-Indiana Sea Grant, Purdue University, West Lafayette, Indiana, United States of America; 3 School of Freshwater Sciences, University of Wisconsin-Milwaukee, Milwaukee, Wisconsin, United States of America; 4 Lake Michigan Biological Station, Illinois Natural History Survey, University of Illinois, Zion, Illinois, United States of America; 5 Department of Environmental Science and Ecology, The College at Brockport-State University of New York, Brockport, New York, United States of America; University of Shiga Prefecture, JAPAN

## Abstract

In aquatic systems, food web linkages are often assessed using diet contents, stable isotope ratios, and, increasingly, fatty acid composition of organisms. Some correlations between different trophic metrics are assumed to be well-supported; for example, particular stable isotope ratios and fatty acids seem to reflect reliance on benthic or pelagic energy pathways. However, understanding whether the assumed correlations between different trophic metrics are coherent and consistent across species represents a key step toward their effective use in food web studies. To assess links among trophic markers, we compared relationships between major diet components, fatty acids, and stable isotope ratios in three fishes: yellow perch (*Perca flavescens*), round goby (*Neogobius melanostomus*), and spottail shiner (*Notropis hudsonius*) collected from nearshore Lake Michigan. Yellow perch and spottail shiner are native in this system, while round goby are a relatively recent invader. We found some evidence for agreement between different trophic metrics, especially between diet components, n-3:n-6 fatty acid ratios, and stable isotope ratios (δ^13^C and δ^15^N). However, we also observed significant variation in observed relationships among markers and species, potentially due to taxonomic variation in the specific diet items consumed (e.g., chydorid microcrustaceans and *Dreissena* mussels) and species-specific biochemical processes. In many of these latter cases, the invasive species differed from the native species. Understanding the effects of taxonomic variation on prey and predator signatures could significantly improve the usefulness of fatty acids in food web studies, whereas diet contents and stable isotopes appear to be reliable indicators of trophic niche in aquatic food webs.

## Introduction

Identifying how energy flows through food webs can help ecologists and natural resource managers assess the adaptability of food webs to environmental change, understand how to best support overall food web structure, and ultimately improve ecosystem resilience [1,2]. Various trophic metrics can be used to elucidate linkages between communities that occupy different trophic levels [3], assess the relative importance of basal energy sources [4–6], and identify key, and different, sources of nutrients supporting ecosystems (e.g., [7]). Some of the more popular methods of assessing trophic position in a food web include traditional diet (gut) content analysis (e.g., [8]), analysis of stable isotope ratios, most often δ^13^C and δ^15^N (e.g., [7]), and assessment of fatty acid composition of organisms (e.g., [9,10]). While it may be assumed that trophic metrics that reveal similar patterns are correlated within an individual as well as consistent across species and systems, explicit assessment of these correlations could lead to improved assumptions about their informative value given that may they operate over different time scales and may vary with diet quality [11,12].

Diet content analysis, either of the guts of dead organisms [13] or analysis of regurgitated items [14], allows for a very straightforward generation of a food web, as researchers literally identify who is eating whom. While it reflects some of what has been consumed over the past few hours or days, regurgitation and variance in the digestibility or assimilation of different items may lead to pronounced over- or under-estimations of the importance of particular diet components to an organism (e.g., [15]). In addition, high individual, temporal, and spatial variability in feeding patterns suggests researchers may need to collect specimens multiple times a year, and over multiple years, to truly identify which organisms are most important to overall food web structure or determine temporal variation in individual or population niches [16].

Stable isotope ratios of animal tissue are another trophic metric used to evaluate food web structure. The use of δ^15^N and a corresponding conversion to trophic factor [17] is popular for assessing trophic position within a food web, while δ^13^C is understood to be a relatively reliable indicator of different primary production sources in aquatic systems (e.g., [5,18]). Overall, stable isotope ratios likely reflect integration of diet items over a span of weeks to months depending on individual growth rates and seasons [19,20]. This may lead to a more complete picture of food web linkages in some respects. However, the ability to discern individual food web components relies on adequate collection and quantification of the isotopic signatures of additional food web members and prey items (which may not be feasible in all studies), as well as distinct isotopic signatures among prey (which may not be consistent in nature; [21,22]). In addition, stable isotope ratios are not completely determined by diets; factors such as spatial variation in underlying trophic processes or geology can influence isotopic signatures of consumers, complicating interpretations across taxa or locations [7,23].

The fatty acid composition of organisms is a potentially powerful trophic metric that reflects what has been incorporated from an individual’s diet over a time span of a few weeks (e.g., [24]); however, given that fatty acid synthesis and metabolism may vary among taxa and individuals, direct interpretations are unclear [25]. In freshwater systems, essential fatty acids that fish are unable to synthesize, such as the polyunsaturated fatty acids *α*-linolenic acid (ALA; 18:3n-3) and arachidonic acid (ARA; 20:4n-6) can be traced from prey to predator to elucidate food web linkages [26]. Fatty acids can also be combined into more comprehensive summary indices, such as the total number of omega-3 (n-3) fatty acids, or the ratios of docosahexaenoic acid (DHA; 22:6n-3) to eicosapentaenoic acid (EPA; 20:5n-3) or total n-3 to n-6 fatty acids. The ratio of n-3 to n-6 in particular has been found to influence organismal fitness and may help identify the dominant energetic pathways on which particular organisms rely [27]. As with stable isotope ratios, individual fatty acid profiles may be influenced by factors unrelated to diet, such as stress level, nutritional status (i.e., well-fed or starving), or growth rate [25,26,28]. There may also be significant taxonomic variation in how fatty acids are synthesized and metabolized *in vivo*. For example, fatty acid synthesis and decomposition pathways vary between freshwater and marine organisms due to adaptations to different fatty acid availability between environments [26,29], where marine fishes generally lack the ability of freshwater fishes to synthesize long-chain polyunsaturated fatty acids like EPA, DHA, and ARA. In addition, these complex interactions among fatty acid intake, synthesis, and metabolism produce a highly multivariate potential indicator of an individual’s position in the food web, and quantitative methods designed to handle the multivariate nature of fatty acid information may be more informative measures of trophic niche than univariate examinations of specific compounds. However, assessments of the relationship between multivariate fatty acid patterns in food webs and other indicators (i.e., diet contents or stable isotopes) and their informative value beyond measures based on singular fatty acids are lacking.

All three of the analytical methods described above are used in food web studies to draw conclusions or make inferences, e.g., identifying which underlying energy source supports a particular population, or what the relative importance of different habitat types are to a given organism. Understanding whether trophic metrics developed via different methodologies are correlated within individual wild-collected organisms in a coherent and consistent way is an important step toward their effective use in food web studies. Though there have been some assessments of the agreement between different metrics in an individual aquatic organism, e.g., assessing how well stable isotope ratios or fatty acid composition of fish tissue reflect diet contents, or how food type influences tissue turnover rates [11,19,25], most such studies have been performed in controlled laboratory settings using simplified diet compositions (but see [15]). In the wild, individuals have access to myriad potential trophic niches and diet items, and temporal and spatial variation in prey availability, consumer isotope ratios, and fatty acid composition may complicate any observed patterns. In addition, given that fatty acid use as a trophic marker is expanding, understanding the relative consistency of any relationships between fatty acids and other well-established and better understood trophic markers may help researchers interpret fatty acid data.

In an effort to better understand relationships among different trophic indicator metrics analyzed for a given animal, we conducted a case study using three fish species collected from Lake Michigan, one of the largest Laurentian Great Lakes in North America, to evaluate the following questions:

1 For multiple trophic metrics measured on the same individual, are there relationships between metrics thought to represent similar energy pathways?
2 Are these relationships consistent among species?

In addition, given the relative lack of understanding of how fatty acid signatures help position an individual within an aquatic food web, we also considered the full suite of fatty acids using multivariate techniques and assessed:

3 Does multivariate consideration of fatty acids offer a more informative analysis of trophic niche compared to univariate comparisons?

We expected that, by measuring multiple trophic markers on the same individual from an aquatic system (large lake), we would see strong relationships between previously-known indicators of reliance on benthic and pelagic pathways (e.g., δ^13^C, EPA, DHA, ARA) and diet items typically found in benthic (e.g., Chironomidae larvae) or pelagic zones (e.g., microcrustacean zooplankton), respectively (Table 1). If this were the case, it would confirm assumptions about the informative value of different trophic markers across species, which, to this point, have rarely been specifically quantified through multiple comparisons in the wild. Alternatively, should relationships between trophic metrics vary among species or markers, this would suggest that significant care should be taken in the interpretation of food web structure in the absence of comprehensive, species-specific marker analyses or sufficient temporal resolution of trophic niches defined by each metric [12].

**Table 1:** Key components of the three trophic metrics that were compared, pairwise, within an individual fish.

Metric	Key component included in analyses (* see Table 2)	Expected to be positively correlated with
Diet contents	Proportion chironomid larvae	Benthic reliance
	Proportion microcrustacean*	Pelagic reliance, Inverse trophic level
	Proportion pelagic*	Pelagic reliance, Inverse trophic level
	Proportion benthic*	Benthic reliance
Fatty acids	18:3n-3 (ALA)	Benthic reliance[9,30]; *Dreissena*[31]
	20:4n-6 (ARA)	Benthic/terrestrial reliance[30, 32]; Macroinvertebrates[32]
	22:6n-3 (DHA)	Pelagic reliance[31]; Copepoda[34]
	20:5n-3 (EPA)	Pelagic reliance[31], Microcrustacean[9, 35, 36]
	n-3:n-6	Benthic reliance[31, 33, 35]
Stable isotopes	δ^13^C	Benthic reliance[5]
	δ^15^N	Trophic level[7]

## Materials and methods

Fish collection and processing methods are described in detail in [37–39]. Briefly, fish were collected in summer and fall of 2010 via 2-hour micromesh gill net sets in 2 to 15 m of water and stored frozen until processing. Fish were collected from 10 different sites (clockwise around the lake from northeast-most site, all sites in United States of America: Arcadia, MI; Muskegon, MI; Saugatuck, MI; Michigan City, IN; Calumet, IN; Highland Park, IL; Dead River, IL; Whitefish Bay, WI; Fox Point, WI; and Sturgeon Bay, WI; see Fig 1 in [39] and S1 Table). Fish specimens for this project were collected and euthanized by multiple field crews, in accordance with standards set out by 3 different institutions. Methods were generally consistent across institutions, with slight differences in the amount of MS-222 required for overdose (see below). Protocols were approved by the Purdue University Animal Care and Use Committee (protocol number 1112000400), the Illinois Institutional Animal Care and Use Committee (protocol number 11185), the University of Wisconsin-Milwaukee IACUC (protocols number 16–16#28 and 08–09 #41). After initial counts, the fish being kept will be anesthetized with an overdose of MS-222 (50–100 mg/L) and then flash frozen on dry ice. Once back at the laboratory the fish were be placed into -80 degree C freezer until ready for shipping. Field collection permits were also obtained from all state Departments of Natural Resources.

**Fig 1:**
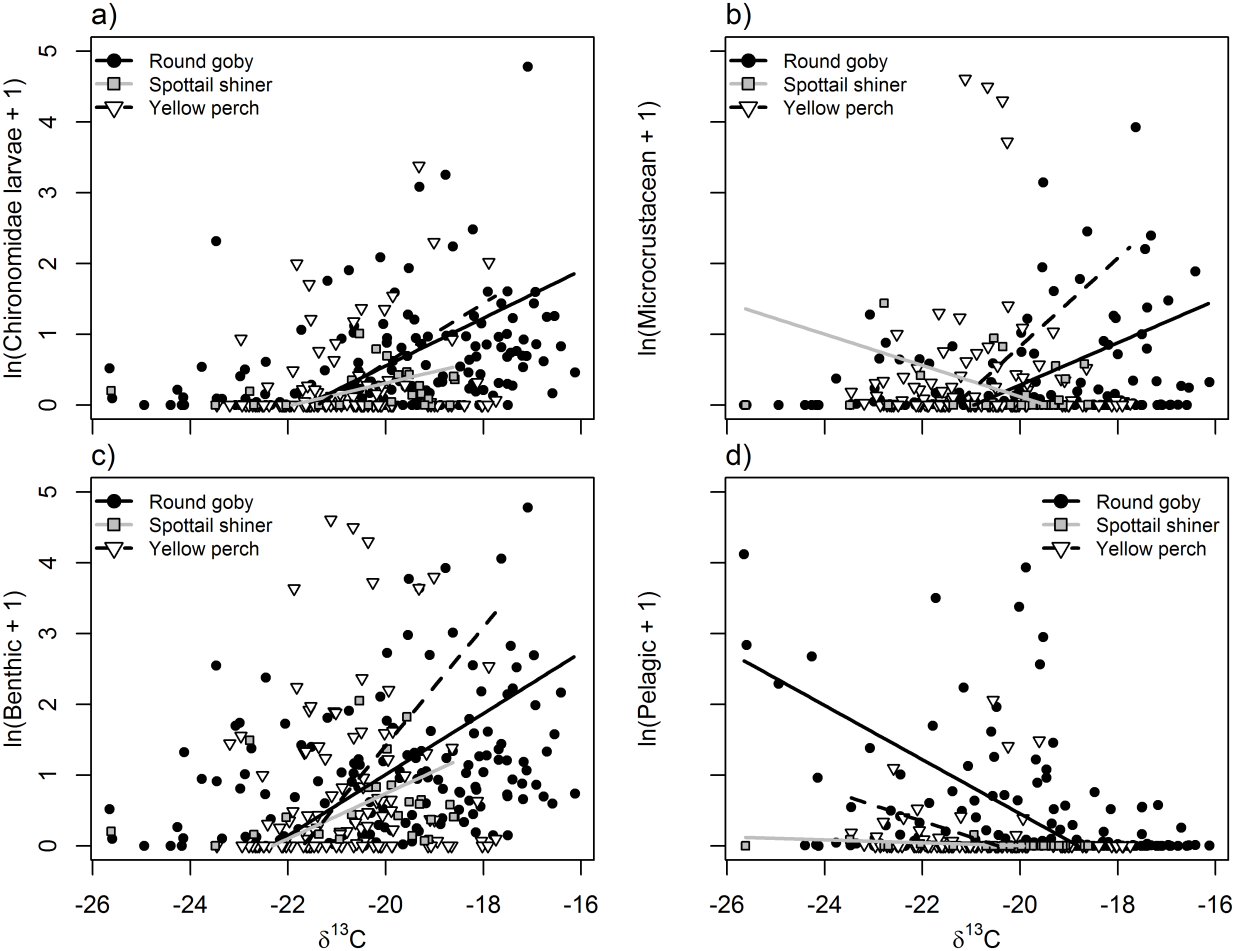
Relationships between δ^13^C values and natural log + 1-transformed biomass (mg) of diet content components a) Chironomidae larvae, b) microcrustaceans, c) benthic-derived resources, and d) pelagic-derived resources. Points and lines represent individual fish and modeled relationships for round goby (black circles, solid black line), spottail shiner (gray boxes, gray line), and yellow perch (white triangles, dashed black line). Species-specific slopes were significantly different in each comparison, where round goby exhibited significant relationships in panels a, c, and d, but no other species exhibited significant relationships; see Table 3.

The collection sites vary in local characteristics such as slope, nearby tributary input, and substrate composition, enabling us to assess relationships between trophic markers across individuals experiencing a range of environmental conditions. By including fish collected from a range of sizes and multiple nearshore locations in Lake Michigan across two seasons, we were able to include a wide range of variation in trophic marker values in this study. Previous studies in this system have suggested that there is minimal seasonal variation in diet contents for these species [35–37], thus we expected that potential for differences in trophic markers attributable to different tissue turnover rates between methods would be minimized. At the same time, these studies suggest spatial variation does exist, which facilitated our ability to evaluate the consistency and power of each trophic marker to capture variability in individual trophic behavior.

To develop correlations between trophic metrics within an individual, each fish included in the present study had at least two different metrics assessed. Fishes that were stored frozen at -20°C before processing were assessed for stable isotopes and diet contents only. Fishes stored frozen at -80°C were assessed for fatty acid profiles and one (or both) of stable isotope ratios and diet contents. Total length and weight of each fish (S1 Table) were recorded before removing stomachs of spottail shiner (*Notropis hudsonius*, STS) and yellow perch (*Perca flavescens*, YEP), and entire digestive tracts of round goby (*Neogobius melanostomus*, ROG). Diet contents were assessed as described in [37–39]. Briefly, individual diet items were identified to lowest taxonomic level feasible, photographed, and measured. Whole diet contents were dried and weighed, and the measured lengths were used to estimate total biomass of a given prey taxon via published length-weight equations. After digestive tracts were removed, fish were homogenized and dried homogenate was analyzed for fatty acids and δ^13^C and δ^15^N. When possible, all three metrics were assessed for an individual; however, small body sizes of some fish precluded assessment of all 3 metrics on each fish. Because all three analyses were not performed on every individual fish, three different datasets were developed for each pairwise combination of trophic metrics (e.g., cases where at least two analyses were performed on an individual): diet-fatty acid, fatty acid-stable isotope, and diet-stable isotope. Diet data were expressed as total biomass (mg) of each item type and fatty acids were expressed as a proportion of the total mass of all fatty acids measured. δ^13^C values were corrected for individual lipid content using species- and system-specific mathematical corrections [40].

Though fish used in these analyses were of different sizes and were collected across different sites and seasons, the power of the current study is in assessing the potential associations of multiple trophic markers within an individual without an understanding of which external factors are shaping those patterns. Though certainly preferred, additional information such as isotopic or fatty acid signatures of diet contents or potential prey is not always available in a given study. We assessed relationships between key components of diet contents, fatty acids, and stable isotopes within individual fish, specifically identifying components that are thought to integrate information about the trophic niche and energy sources supporting development of individuals (Table 1). Fatty acid and stable isotope data followed normal distributions, whereas diet content data were natural log + 1—transformed to approximate a normal distribution. Because we were interested in identifying and comparing relationships between trophic metrics that were estimated with error, and there was no clear reason to assign any variables as predictor or response as in linear regression, we used standardized major axis regression [41] to evaluate relationships between trophic markers using R package ‘smatr’[42]. Standardized major axis regression minimizes bivariate residuals by minimizing the area of triangles formed by pairs of observations and the predicted regression line, thereby accounting for errors in both y and x variables and will estimate the same relationship between variables regardless of their order in the model. For each relationship between trophic markers, we first tested for equal slopes among species using a χ^2^ test comparing models including and excluding species-specific slopes [42]. When this test was significant (i.e., slopes significantly differed among species), separate slopes were estimated for each species. When this test was not significant (i.e., slopes did not differ among species), a single slope was estimated. The mean and 95% confidence intervals of species-specific slopes were used to compare relationships among species, and species-specific Pearson correlation tests were used to examine whether trophic markers were significantly correlated within species. All analyses were performed in program R version 3.4.3 [43].

The five fatty acid components included in the above analyses (Table 1; ALA, ARA, DHA, EPA, and the ratio of n-3:n-6 fatty acids), are thought to differentiate between energetic pathways in aquatic systems (e.g., [9,44]) and are relatively commonly used to assess food web structure (reviewed in [45]). At the same time, focusing on single fatty acids or ratios ignores the highly multivariate and co-dependent nature of organismal fatty acid signatures due to metabolic and synthesis pathways [28,29]. A more holistic, multivariate indicator of fatty acid composition could be better related to indicators of diet or isotopic niche than single fatty acids alone [45], similar to the idea of assessing the cumulative consumption of benthic or pelagic prey items [18]. To this end, we created orthogonal indicators of individual fatty acid composition using principal components analysis (PCA) on all 28 fatty acids identified across species in each of the pairwise datasets including fatty acids (i.e., one PCA each for the fatty acid-diet and fatty acid-stable isotope datasets). For each PCA, the first three principal components (PCs) had the greatest relative explanatory power compared to following PCs and were easily interpreted, i.e., loadings of individual fatty acids were clear and made ecological sense (see Results and S2 Table). We then related these multivariate indicators of fatty acid composition to diet and isotopic indicator components following the modeling methods described above. Due to the large number of models (N = 56) created during this study, we sought to control our error rate by only considering relationships as significant at a Holm-corrected *α* = 0.05 for 56 comparisons, meaning a *P* < 0.0009 was considered significant.

## Results

### Summary

Twenty-seven unique diet content categories were identified across the three fish species sampled. In turn, these were grouped into main components to be included in analyses (Table 2). In total, we identified 30 comparisons between trophic metrics (out of 56) where one species exhibited a significant correlation between markers. Of these, there were eight comparisons where at least two species exhibited significant correlations (Tables 3, 4 and 5), and only one case where trophic metrics were significantly correlated in the same direction across all three fish species. While there were many instances where slopes of these relationships differed significantly among species, delving further, the species-specific correlations themselves were not always significant. Below, we primarily focus on comparisons where at least one species-specific correlation between markers was significant, as we deem these instances most ecologically relevant to the objective of our study.

**Table 2:** Mean biomass (mg) of each diet category for each species included in analysis (ROG = round goby, STS = spottail shiner, YEP = yellow perch). Prey item signifies the lowest taxonomic resolution to which a prey item was identified, which were then grouped into larger taxonomic and energy source categories for analysis.

Prey item	Diet category	Energy source	ROG	STS	YEP
Acari	Other	Benthic	0.014	0.041	0.000
Amphipoda	Other	Benthic	0.104	0.000	0.001
Chironomidae larvae	Chironomidae larvae	Benthic	2.057	0.323	0.735
Chironomidae pupae	Other	Benthic	0.320	0.217	1.588
Chydoridae	Microcrustacean	Benthic	1.507	0.338	4.036
Decapoda	Other	Benthic	0.139	0.000	67.100
Eggs	Other	Benthic	0.276	0.000	0.000
Gastropoda	Other	Benthic	0.005	0.000	<0.0001
Harpacticoida	Microcrustacean	Benthic	0.005	0.000	0.000
Hydracarina	Other	Benthic	0.000	0.000	0.056
Isopoda	Other	Benthic	0.298	0.000	0.129
Nematoda	Other	Benthic	0.013	0.000	0.000
Odonata	Other	Benthic	0.000	0.234	0.000
Oligochaeta	Other	Benthic	0.005	0.000	0.000
Ostracoda	Other	Benthic	0.104	0.000	0.008
Sphaeriidae	Other	Benthic	0.015	<0.0001	0.000
Bosminidae	Microcrustacean	Pelagic	<0.0001	0.000	0.010
Cercopagidae	Microcrustacean	Pelagic	0.000	0.000	0.034
Unidentified Copepoda	Microcrustacean	Pelagic	0.000	0.000	0.005
Cyclopoida	Microcrustacean	Pelagic	0.006	0.001	0.000
*Daphnia*	Microcrustacean	Pelagic	<0.0001	0.000	0.000
*Dreissena*	Other	Pelagic	1.891	0.010	0.009
*Mysis*	Other	Pelagic	0.000	0.000	0.129
Nauplii	Microcrustacean	Pelagic	<0.0001	0.000	0.000
Other zooplankton	Microcrustacean	Pelagic	0.000	0.000	<0.0001
Veliger	Other	Pelagic	0.001	0.000	0.000
Coleoptera	Other	Terrestrial	0.000	0.047	0.000
Hymenoptera	Other	Terrestrial	0.000	0.173	0.000
Fish	Fish	Fish	0.000	0.000	5.062

**Table 3:** Standard major axis regression results (mean and 95% confidence intervals for species-specific intercepts and slopes) for pairwise relationships between key diet components and stable isotope ratios within individual fish, with associated test statistics and P-value for likelihood ratio test (LR; χ^2^, 2 d.f.) for equality of slopes bolded and italicized when significant. Species-specific relationships are bolded and italicized when a within-species correlation test was significant; differences among species were assessed via overlapping confidence intervals. See visualization of significant relationships in Fig 1. Significance was determined at a corrected P < 0.0009.

Isotope	Diet item	ROG		STS		YEP		LR	P
		Intercept	Slope	Intercept	Slope	Intercept	Slope		
δ^13^C	Chironomidae larvae	***7*.*30*** ***(6*.*26*, *8*.*34)***	***0*.*34*** ***(0*.*29*, *0*.*39)***	3.56 (2.14, 4.97)	0.16 (0.11, 0.25)	9.04 (7.39, 10.69)	0.42 (0.35, 0.51)	***16*.*64***	***0*.*0002***
δ^13^C	Microcrustacean	6.26 (5.30, 7.21)	0.30 (0.25, 0.35)	-4.29 (-6.24, -2.35)	-0.22 (-0.33, -0.15)	13.31 (10.84, 15.79)	0.62 (0.52, 0.75)	***40*.*92***	***0*.*0000***
δ^13^C	Pelagic	***-7*.*20*** ***(-8*.*37*, *-6*.*02)***	***-0*.*38*** ***(-0*.*45*, *-0*.*33)***	-0.41 (-0.58, -0.23)	-0.02 (-0.03, -0.01)	-4.60 (-5.49, -3.71)	-0.22 (-0.27, -0.19)	***109*.*34***	***0*.*0000***
δ^13^C	Benthic	***9*.*63*** ***(8*.*30*, *10*.*97)***	***0*.*43*** ***(0*.*37*, *0*.*50)***	7.11 (4.30, 9.92)	0.32 (0.21, 0.48)	18.15 (14.81, 21.48)	0.84 (0.69, 1.01)	***34*.*11***	***0*.*0000***
δ^15^N	Chironomidae larvae	9.12 (7.72, 10.51)	-0.99 (-1.16, -0.84)	-3.20 (-4.68, -1.73)	0.38 (0.25, 0.57)	4.52 (3.71, 5.33)	-0.47 (-0.57, -0.39)	***42*.*11***	***0*.*0000***
δ^15^N	Microcrustacean	6.93 (6.16, 7.69)	-0.77 (-0.86, -0.69)	7.22 (6.34, 8.10)	-0.77 (-0.86, -0.69)	7.24 (6.43, 8.06)	-0.77 (-0.86, -0.69)	7.35	0.0253
δ^15^N	Pelagic	-9.26 (-10.82, -7.70)	1.12 (0.96, 1.32)	0.45 (0.29, 0.61)	-0.05 (-0.07, -0.03)	2.36 (1.92, 2.79)	-0.25 (-0.30, -0.21)	***217*.*77***	***0*.*0000***
δ^15^N	Benthic	10.26 (9.17, 11.36)	-1.07 (-1.21, -0.95)	10.36 (9.10, 11.63)	-1.07 (-1.21, -0.95)	10.36 (9.20, 11.52)	-1.07 (-1.21, -0.95)	9.73	0.0077

**Table 4:** Standard major axis regression results (mean and 95% confidence intervals for species-specific intercepts and slopes) for pairwise relationships between fatty acids and key diet components within individual fish, with associated test statistics and P-value for likelihood ratio test (LR; χ^2^, 2 d.f.) for equality of slopes bolded and italicized when significant. Species-specific relationships are bolded and italicized when a within-species correlation test was significant at corrected P < 0.0009; differences among species were assessed via overlapping confidence intervals. See visualization of significant relationships in Figs 2, 3 and 5.

Fatty acid	Diet item	ROG		STS		YEP		LR	P
		Intercept	Slope	Intercept	Slope	Intercept	Slope		
ALA	Chironomidae larvae	-1.00 (-1.23, -0.76)	68.85 (60.76, 78.01)	0.95 (0.61, 1.28)	-38.12 (-57.42, -25.31)	-0.49 (-0.64, -0.34)	34.18 (29.86, 39.12)	***56*.*18***	***0*.*0000***
ALA	Microcrustacean	-1.00 (-1.17, -0.84)	58.63 (53.61, 64.22)	-0.88 (-1.17, -0.59)	58.63 (53.61, 64.22)	-0.99 (-1.23, -0.76)	58.63 (53.61, 64.22)	6.40	0.0408
ALA	Pelagic	-1.21 (-1.43, -0.98)	65.48 (57.75, 74.25)	-0.08 (-0.12, -0.04)	4.81 (3.30, 7.01)	-0.34 (-0.43, -0.26)	18.20 (15.86, 20.88)	***236*.*97***	***0*.*0000***
ALA	Benthic	-0.76 (-0.99, -0.54)	78.62 (71.78, 86.18)	-0.87 (-1.26, -0.47)	78.62 (71.78, 86.18)	-0.96 (-1.26, -0.65)	78.62 (71.78, 86.18)	9.34	0.0094
ARA	Chironomidae larvae	***3*.*02*** ***(2*.*70*, *3*.*34)***	***-41*.*58*** ***(-46*.*96*, *-36*.*82)***	-0.79 (-1.23, -0.34)	20.90 (14.05, 31.09)	***1*.*74*** ***(1*.*52*, *1*.*95)***	***-24*.*36*** ***(-27*.*79*, *-21*.*34)***	***38*.*24***	***0*.*0000***
ARA	Microcrustacean	***2*.*57*** ***(2*.*35*, *2*.*78)***	***-37*.*92*** ***(-41*.*41*, *-34*.*81)***	2.07 (1.74, 2.40)	-37.92 (-41.41, -34.81)	***2*.*61*** ***(2*.*33*, *2*.*89)***	***-37*.*92*** ***(-41*.*41*, *-34*.*81)***	2.59	0.2745
ARA	Pelagic	-1.99 (-2.31, -1.68)	39.55 (34.87, 44.86)	-0.12 (-0.18, -0.06)	2.64 (1.76, 3.95)	-0.69 (-0.81, -0.57)	12.97 (11.31, 14.87)	***202*.*20***	***0*.*0000***
ARA	Benthic	***4*.*02*** ***(3*.*73*, *4*.*31)***	***-50*.*83*** ***(-55*.*52*, *-46*.*64)***	3.08 (2.61, 3.55)	-50.83 (-55.52, -46.64)	3.89 (3.55, 4.24)	-50.83 (-55.52, -46.64)	3.05	0.2172
ARA	Chironomidae larvae	***-2*.*57*** ***(-2*.*87*, *-2*.*26)***	***20*.*86*** ***(19*.*09*, *22*.*81)***	-1.72 (-1.99, -1.46)	20.86 (19.09, 22.81)	-2.06 (-2.30, -1.83)	20.86 (19.09, 22.81)	0.15	0.9288
ARA	Microcrustacean	-2.64 (-3.03, -2.24)	19.73 (17.42, 22.34)	2.75 (1.66, 3.84)	-27.03 (-40.67, -17.97)	***-3*.*29*** ***(-3*.*79*, *-2*.*78)***	***32*.*32*** ***(28*.*35*, *36*.*85)***	***28*.*52***	***0*.*0000***
ARA	Pelagic	***3*.*30*** ***(2*.*92*, *3*.*68)***	***-19*.*69*** ***(-22*.*24*, *-17*.*42)***	-0.22 (-0.32, -0.13)	2.50 (1.68, 3.70)	-1.20 (-1.39, -1.02)	11.31 (9.86, 12.97)	***89*.*42***	***0*.*0000***
ARA	Benthic	***-3*.*03*** ***(-3*.*55*, *-2*.*52)***	***26*.*99*** ***(23*.*92*, *30*.*44)***	4.27 (2.74, 5.79)	-39.04 (-58.07, -26.24)	***-3*.*98*** ***(-4*.*66*, *-3*.*30)***	***42*.*61*** ***(37*.*26*, *48*.*72)***	***25*.*13***	***0*.*0000***
DHA	Chironomidae larvae	***2*.*14*** ***(1*.*92*, *2*.*36)***	***-19*.*94*** ***(-22*.*50*, *-17*.*67)***	-0.75 (-1.15, -0.35)	8.65 (5.94, 12.60)	***2*.*06*** ***(1*.*81*, *2*.*32)***	***-10*.*96*** ***(-12*.*51*, *-9*.*61)***	***50*.*19***	***0*.*0000***
DHA	Microcrustacean	***1*.*72*** ***(1*.*55*, *1*.*88)***	***-17*.*55*** ***(-19*.*24*, *-16*.*07)***	2.21 (1.86, 2.57)	-17.55 (-19.24, -16.07)	3.19 (2.88, 3.50)	-17.55 (-19.24, -16.07)	5.86	0.0533
DHA	Pelagic	1.78 (1.56, 2.00)	-18.97 (-21.51, -16.72)	0.14 (0.08, 0.19)	-1.09 (-1.64, -0.73)	-0.86 (-1.01, -0.72)	5.84 (5.09, 6.70)	***221*.*42***	***0*.*0000***
DHA	Benthic	***2*.*87*** ***(2*.*66*, *3*.*08)***	***-23*.*38*** ***(-25*.*56*, *-21*.*47)***	3.27 (2.73, 3.80)	-23.38 (-25.56, -21.47)	***4*.*65*** ***(4*.*27*, *5*.*04)***	***-23*.*38*** ***(-25*.*56*, *-21*.*47)***	7.90	0.0192
n-3:n-6	Chironomidae larvae	-2.32 (-2.71, -1.94)	1.18 (1.04, 1.33)	1.62 (1.05, 2.20)	-0.62 (-0.92, -0.42)	-3.76 (-4.32, -3.19)	1.57 (1.37, 1.81)	***22*.*64***	***0*.*0000***
n-3:n-6	Microcrustacean	***-2*.*43*** ***(-2*.*79*, *-2*.*07)***	***1*.*12*** ***(0*.*99*, *1*.*27)***	-1.68 (-2.43, -0.94)	0.84 (0.58, 1.23)	6.54 (5.67, 7.40)	-2.40 (-2.75, -2.09)	***73*.*30***	***0*.*0000***
n-3:n-6	Pelagic	***3*.*09*** ***(2*.*73*, *3*.*45)***	***-1*.*12*** ***(-1*.*27*, *-0*.*99)***	-0.16 (-0.24, -0.09)	0.08 (0.05, 0.12)	2.24 (1.93, 2.54)	-0.84 (-0.96, -0.73)	***95*.*32***	***0*.*0000***
n-3:n-6	Benthic	***-2*.*75*** ***(-3*.*24*, *-2*.*26)***	***1*.*54*** ***(1*.*36*, *1*.*73)***	-2.14 (-3.31, -0.97)	1.22 (0.81, 1.83)	-7.12 (-8.26, -5.98)	3.10 (2.69, 3.56)	***60*.*22***	***0*.*0000***

**Table 5:** Standard major axis regression results (mean and 95% confidence intervals for species-specific intercepts and slopes) for pairwise relationships between stable isotope ratios and fatty acids within individual fish, with associated test statistics and P-value for likelihood ratio test (LR; χ^2^, 2 d.f.) for equality of slopes bolded and italicized when significant. Species-specific relationships are bolded and italicized when a within-species correlation test was significant; differences among species were assessed via overlapping confidence intervals. See visualization of significant relationships in Figs 3 and 4. Significance was determined at a corrected P < 0.0009.

Isotope	Fatty Acid	ROG		STS		YEP		LR	P
		Intercept	Slope	Intercept	Slope	Intercept	Slope		
δ^13^C	ALA	-0.06 (-0.08, -0.05)	0.00 (-0.01, 0.00)	-0.09 (-0.12, -0.06)	-0.01 (-0.01, 0.00)	0.30 (0.25, 0.36)	0.01 (0.01, 0.02)	**71.71**	**0.0000**
δ^13^C	ARA	0.18 (0.16, 0.20)	0.01 (0.00, 0.01)	-0.13 (-0.18, -0.08)	-0.01 (-0.01, -0.01)	-0.31 (-0.38, -0.24)	-0.02 (-0.02, -0.01)	**73.15**	**0.0000**
δ^13^C	EPA	-0.06 (-0.10, -0.03)	-0.01 (-0.01, -0.01)	0.31 (0.25, 0.37)	0.01 (0.01, 0.01)	0.53 (0.45, 0.61)	0.02 (0.02, 0.02)	**31.77**	**0.0000**
δ^13^C	DHA	***0*.*41*** ***(0*.*36*, *0*.*47)***	***0*.*02*** ***(0*.*01*, *0*.*02)***	-0.37 (-0.51, -0.23)	-0.02 (-0.03, -0.02)	-0.74 (-0.91, -0.56)	-0.04 (-0.05, -0.04)	**56.76**	**0.0000**
δ^13^C	n-3:n-6	5.50 (4.87, 6.12)	0.17 (0.14, 0.20)	-2.62 (-4.01, -1.24)	-0.24 (-0.32, -0.19)	8.88 (7.69, 10.07)	0.30 (0.25, 0.37)	**19.02**	**0.0001**
δ^15^N	ALA	***0*.*14*** ***(0*.*13*, *0*.*16)***	***-0*.*01*** ***(-0*.*02*, *-0*.*01)***	0.15 (0.13, 0.16)	-0.01 (-0.02, -0.01)	***0*.*15*** ***(0*.*13*, *0*.*16)***	***-0*.*01*** ***(-0*.*02*, *-0*.*01)***	6.63	0.0364
δ^15^N	ARA	-0.09 (-0.11, -0.07)	0.02 (0.02, 0.02)	-0.12 (-0.14, -0.10)	0.02 (0.02, 0.02)	-0.11 (-0.13, -0.09)	0.02 (0.02, 0.02)	1.84	0.3993
δ^15^N	EPA	0.35 (0.33, 0.37)	-0.03 (-0.03, -0.02)	0.33 (0.30, 0.35)	-0.03 (-0.03, -0.02)	***0*.*34*** ***(0*.*32*, *0*.*37)***	***-0*.*03*** ***(-0*.*03*, *-0*.*02)***	12.21	0.0022
δ^15^N	DHA	***0*.*52*** ***(0*.*47*, *0*.*57)***	***-0*.*05*** ***(-0*.*06*, *-0*.*04)***	0.57 (0.52, 0.63)	-0.05 (-0.06, -0.04)	0.62 (0.57, 0.68)	-0.05 (-0.06, -0.04)	1.34	0.5119
δ^15^N	n-3:n-6	6.93 (6.08, 7.77)	-0.57 (-0.67, -0.48)	6.39 (5.21, 7.58)	-0.45 (-0.59, -0.34)	-0.54 (-1.16, 0.08)	0.34 (0.28, 0.42)	**14.22**	**0.0008**

**Fig 2:**
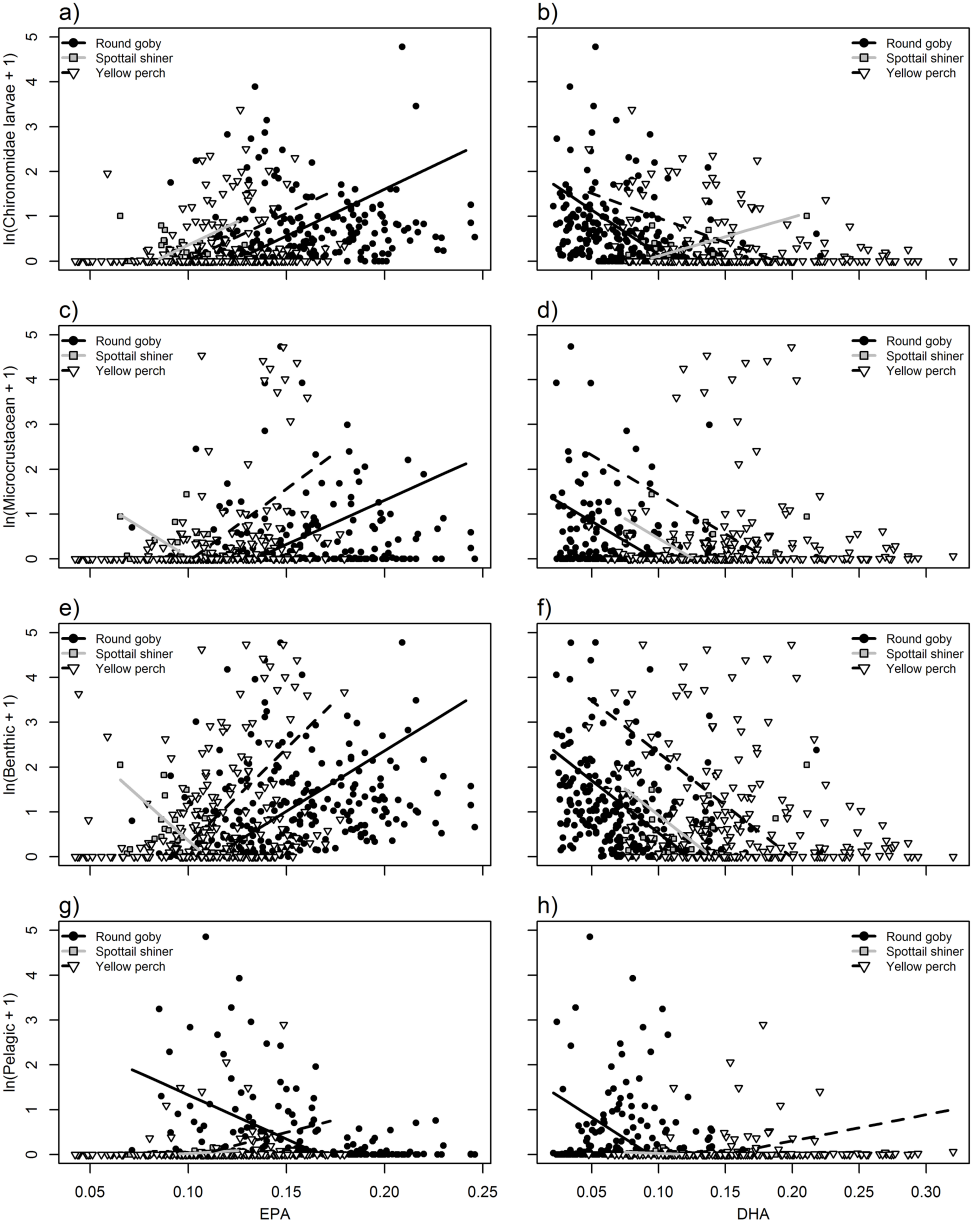
Relationships between EPA (20:5n-3; left column) or DHA (C22:6n-3; right column) and natural log + 1-transformed biomass (mg) of diet content components: Chironomidae larvae (a,b) microcrustaceans (c,d), benthic-derived resources (e,f), and pelagic-derived resources (g,h). Points and lines represent individual fish and modeled relationships for round goby (black circles, solid black line), spottail shiner (gray boxes, gray line), and yellow perch (white triangles, dashed black line). See Table 4 for significance of slope and interaction terms.

**Fig 3:**
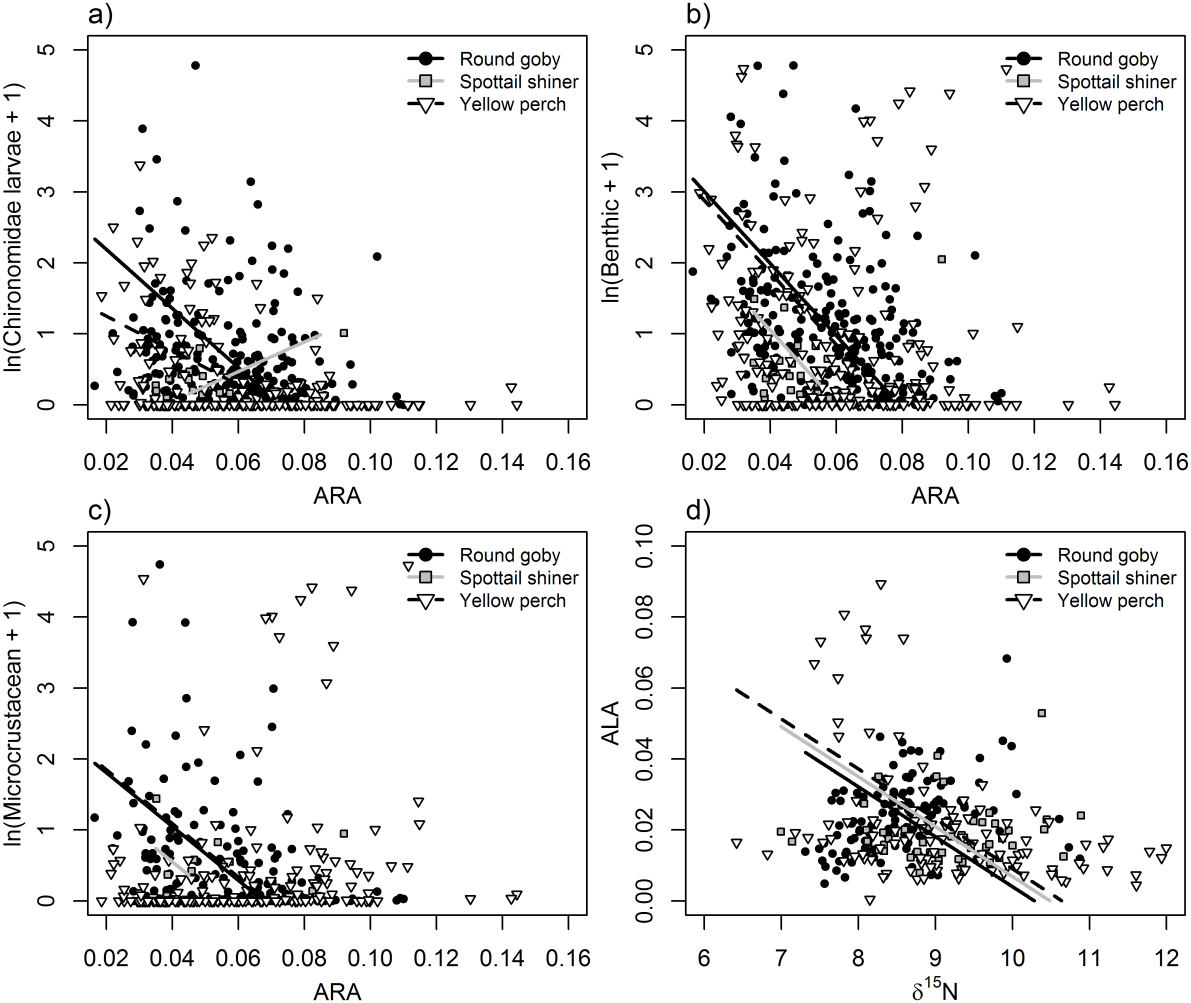
Relationships between ARA (20:4n-6; panels a, b, c) or ALA (20:4n-6; d) and a) natural log + 1-transformed biomass (mg) of Chironomidae larvae, b) benthic-derived resources, c) microcrustaceans, and d) δ^15^N values. Points and lines represent individual fish and modeled relationships for round goby (black circles, solid black line), spottail shiner (gray boxes, gray line), and yellow perch (white triangles, dashed black line). See Tables 4 and 5 for significance of slope and interaction terms.

**Fig 4:**
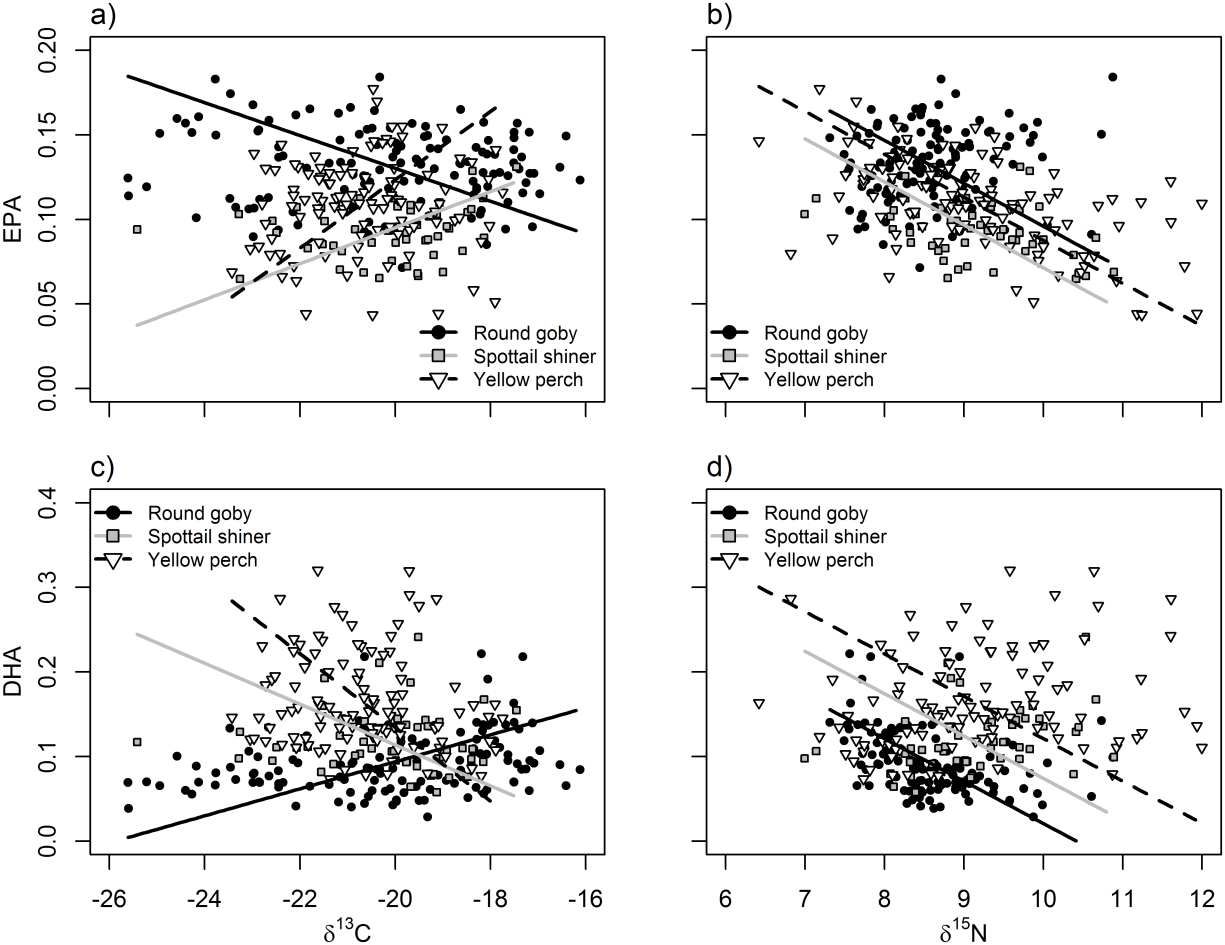
Relationships between δ^13^C values (a,c) or δ^15^N values (b,d) and fatty acids EPA (20:5n-3, a,b) and DHA (22:6n-3, b,d). Points and lines represent individual fish and modeled relationships for round goby (black circles, solid black line), spottail shiner (gray boxes, gray line), and yellow perch (white triangles, dashed black line). See Table 5 for significance of slope and interaction terms.

**Fig 5:**
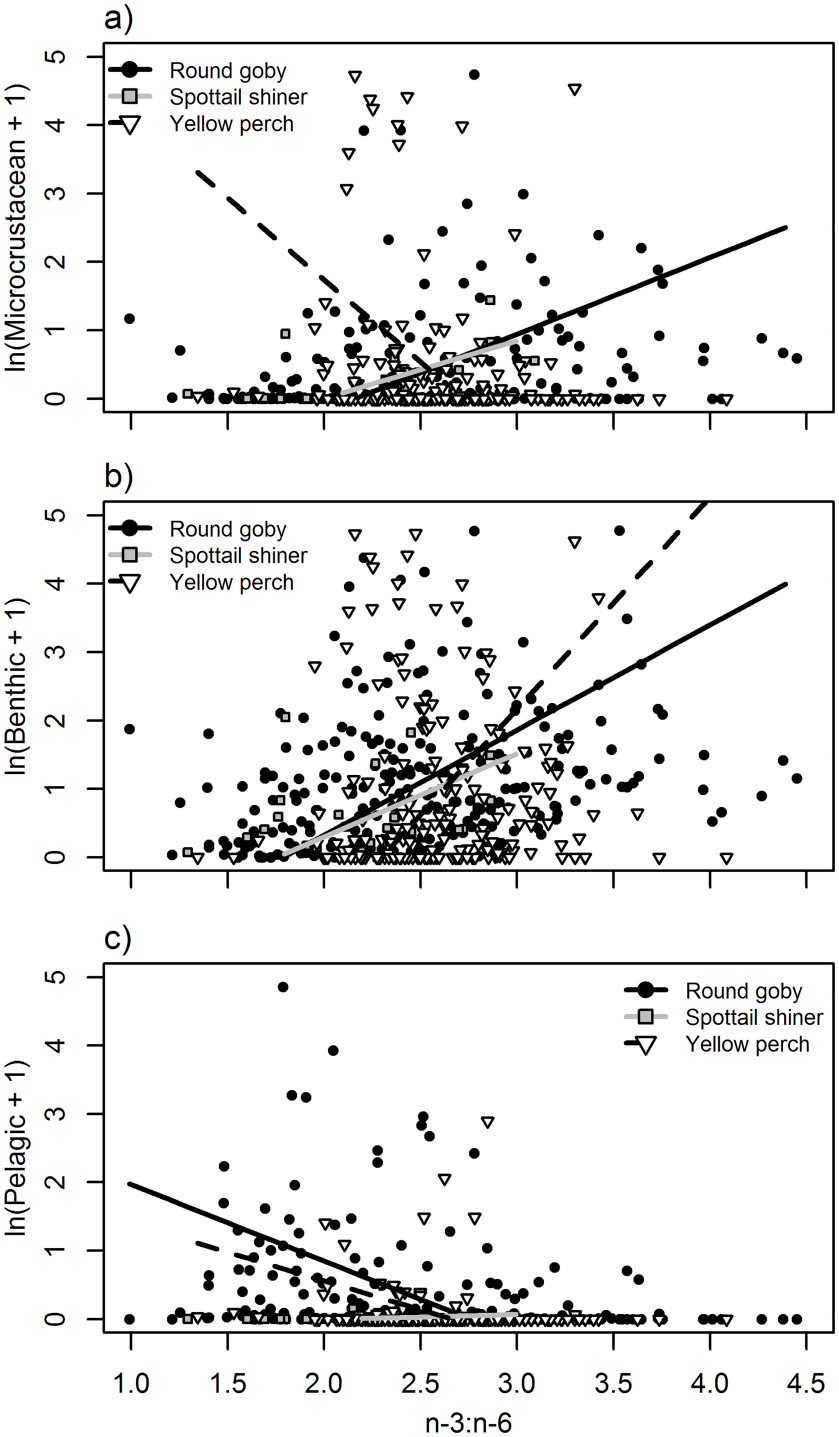
Relationships between n-3:n-6 ratios and natural log + 1-transformed diet item biomass (mg) a) microcrustaceans, b) benthic-derived resources, and c) pelagic-derived resources. Points and lines represent individual fish and modeled relationships for round goby (black circles, solid black line), spottail shiner (gray boxes, gray line), and yellow perch (white triangles, dashed black line). See Table 4 for significance of slope and interaction terms.

**Fig 6:**
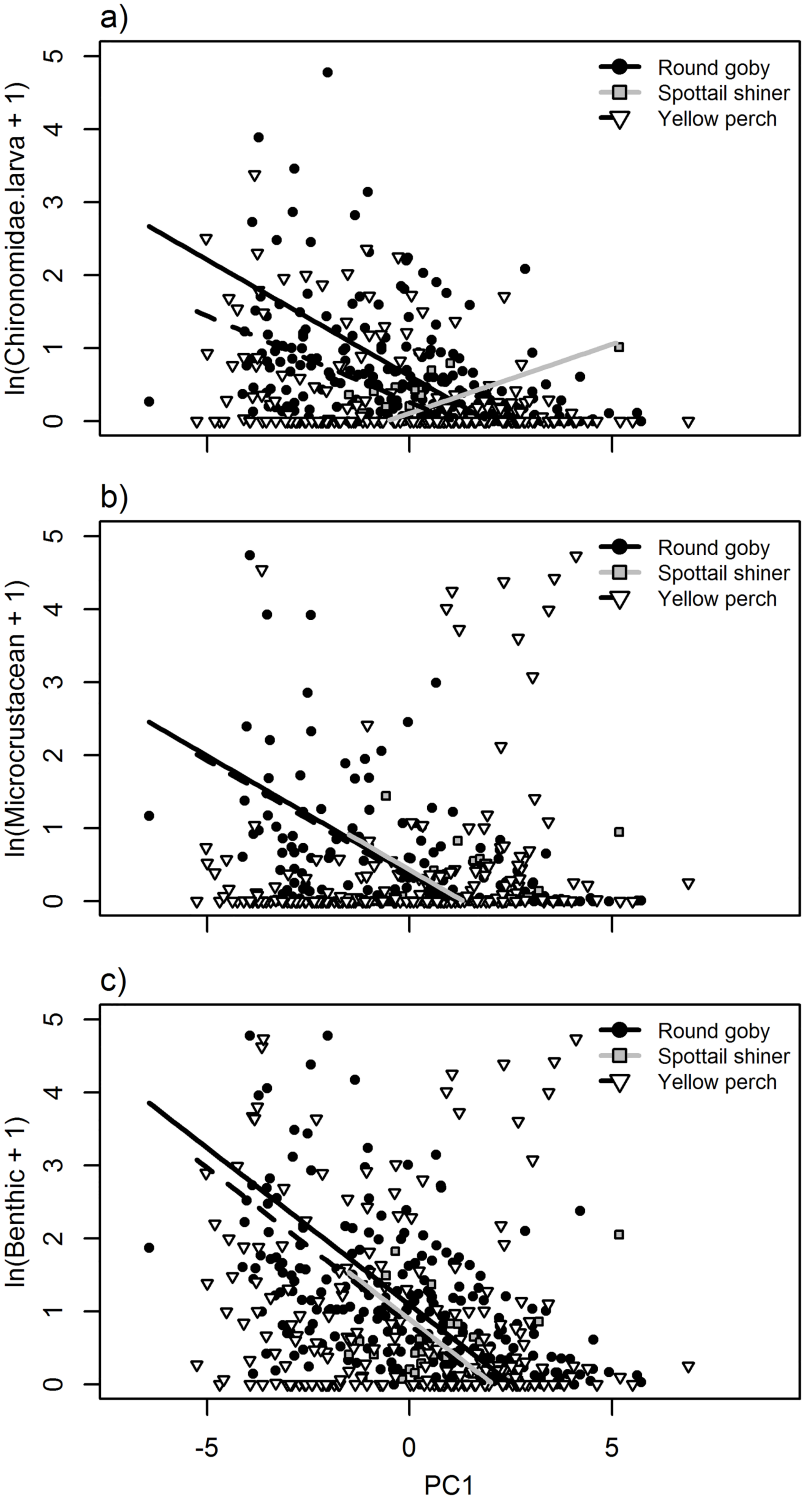
Relationships between fatty acid principal component 1 (PC1) and natural log + 1-transformed diet item biomass (mg) of a) Chironomidae larvae, b) microcrustaceans, and c) benthic-derived resources. Points and lines represent individual fish and modeled relationships for round goby (black circles, solid black line), spottail shiner (gray boxes, gray line), and yellow perch (white triangles, dashed black line). See Table 6 for significance of slope and interaction terms.

**Fig 7:**
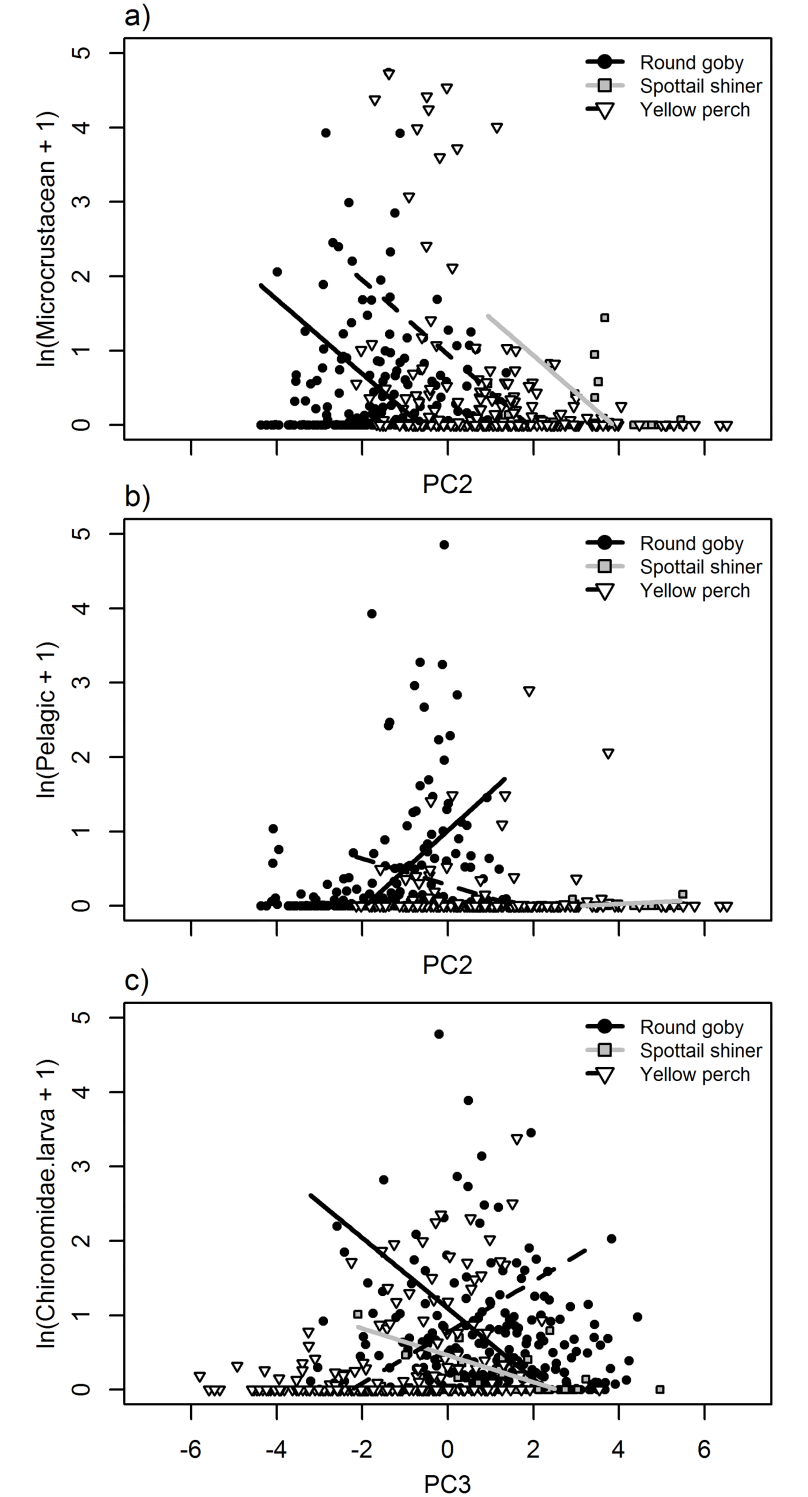
Relationships between fatty acid principal components 2 or 3 (PC2: Panels a, b; PC3: Panel c) and natural log + 1-transformed diet item biomass (mg) of a) microcrustaceans, b) pelagic-derived resources, and c) Chironomidae larvae. Points and lines represent individual fish and modeled relationships for round goby (black circles, solid black line), spottail shiner (gray boxes, gray line), and yellow perch (white triangles, dashed black line). See Table 6 for significance of slope and interaction terms.

**Fig 8:**
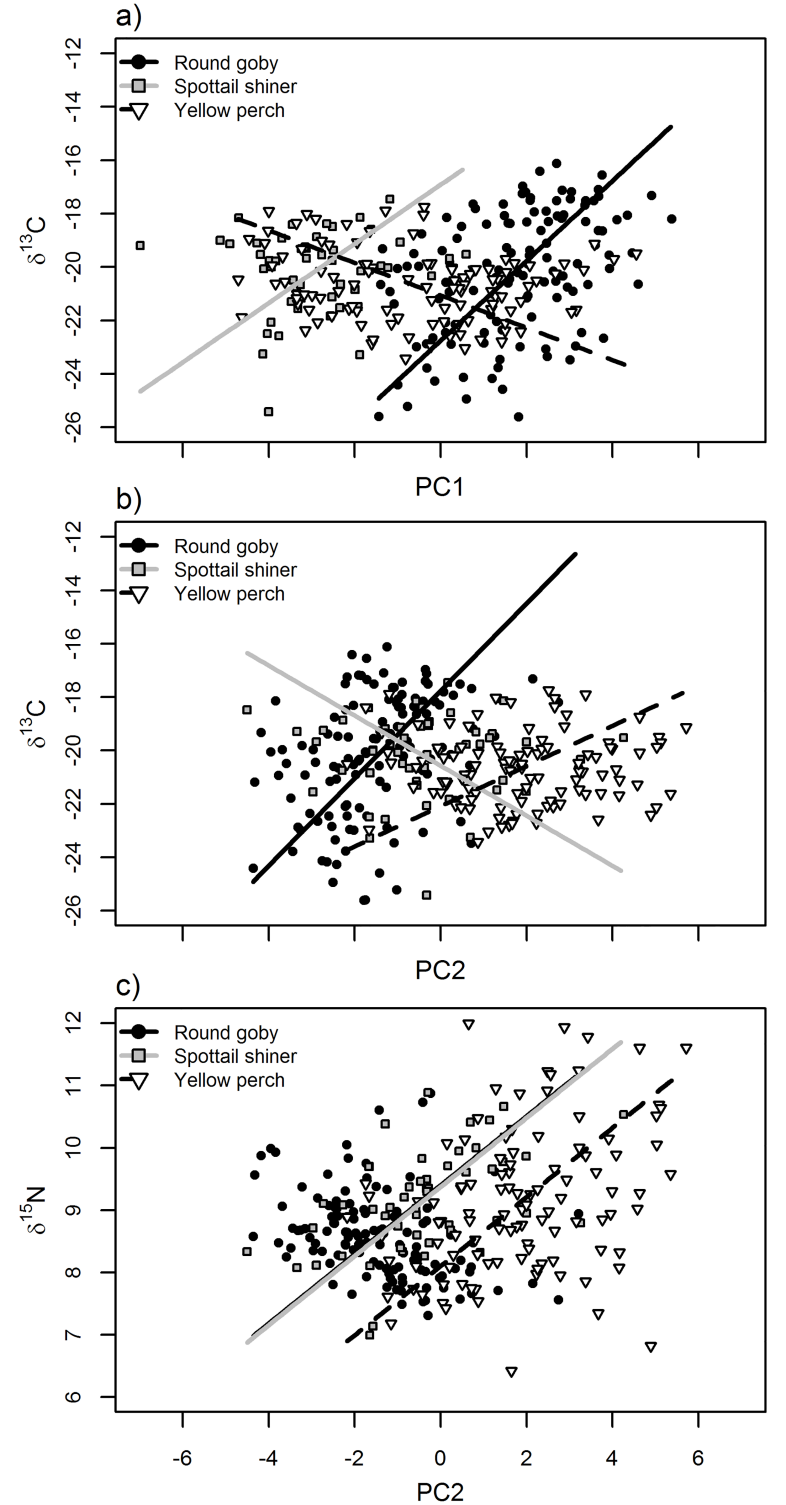
Relationships between fatty acid principal components 1 or 2 (PC1: Panel a; PC2: Panel b,c) and δ^13^C (a,b) and δ^15^N (c). Points and lines represent individual fish and modeled relationships for round goby (black circles, solid black line), spottail shiner (gray boxes, gray line), and yellow perch (white triangles, dashed black line). See Table 6 for significance of slope and interaction terms.

In general, δ^13^C exhibited strong, significant relationships to diet items (Table 3; Fig 1). Diet contents were significantly related to fatty acid signatures of EPA, DHA, and ARA (Table 4; Figs 2 and 3), whereas stable isotopes were primarily related to DHA, EPA and ALA (Table 5; Fig 4). Using n-3:n-6 as a multivariate fatty acid indicator, n-3:n-6 ratios were variably related to some aspects of diet, but not stable isotope values (Tables 4 and 5; Fig 5). Using PCA to develop composite indices of fatty acid composition revealed significant relationships between several principal components and diet items as well as between principal components and both isotopic indicators (Table 6; Figs 7 and 8). All relationships between different markers are summarized in Tables 3, 4, 5 and 6, while significant relationships are depicted in Figs 1, 2, 3, 4, 5, 6, 7 and 8.

**Table 6:** Standard major axis regression results (mean and 95% confidence intervals for species-specific intercepts and slopes) for pairwise relationships between fatty acid principal component axis (PCs) and trophic markers within individual fish, with associated test statistics and P-value for likelihood ratio test (LR; χ^2^, 2 d.f.) for equality of slopes bolded and italicized when significant. Species-specific relationships are bolded and italicized when a within-species correlation test was significant; differences among species were assessed via overlapping confidence intervals. See visualization of significant relationships in Figs 6, 7 and 8. Significance was determined at a corrected P < 0.0009.

FA Axis	Marker	ROG		STS		YEP		LR	P
		Intercept	Slope	Intercept	Slope	Intercept	Slope		
PC1	Chironomidae larvae	***0*.*62*** ***(0*.*52*, *0*.*72)***	***-0*.*32*** ***(-0*.*36*, *-0*.*28)***	0.11 (-0.05, 0.27)	0.19 (0.13, 0.28)	***0*.*26*** ***(0*.*16*, *0*.*36)***	***-0*.*24*** ***(-0*.*27*, *-0*.*21)***	***14*.*74***	***0*.*0006***
PC1	Microcrustacean	***0*.*38*** ***(0*.*28*, *0*.*48)***	***-0*.*32*** ***(-0*.*35*, *-0*.*30)***	0.43 (0.15, 0.71)	-0.32 (-0.35, -0.30)	0.31 (0.13, 0.50)	-0.32 (-0.35, -0.30)	4.88	0.0871
PC1	Pelagic	0.29 (0.17, 0.41)	0.30 (0.27, 0.34)	-0.01 (-0.02, 0.01)	0.02 (0.02, 0.04)	0.10 (0.04, 0.16)	0.13 (0.11, 0.14)	***151*.*94***	***0*.*0000***
PC1	Benthic	***1*.*09*** ***(0*.*97*, *1*.*21)***	***-0*.*43*** ***(-0*.*47*, *-0*.*40)***	0.89 (0.50, 1.28)	-0.43 (-0.47, -0.40)	0.82 (0.61, 1.02)	-0.43 (-0.47, -0.40)	2.17	0.3372
PC1	δ^13^C	***-22*.*76*** ***(-23*.*37*, *-22*.*14)***	***1*.*50*** ***(1*.*27*, *1*.*76)***	-16.91 (-18.03, -15.80)	1.11 (0.83, 1.47)	-21.06 (-21.39, -20.72)	-0.61 (-0.73, -0.50)	***48*.*46***	***0*.*0000***
PC1	δ^15^N	9.47 (9.28, 9.66)	-0.51 (-0.57, -0.45)	7.65 (7.31, 7.99)	-0.51 (-0.57, -0.45)	8.83 (8.52, 9.13)	-0.51 (-0.57, -0.45)	3.63	0.1629
PC2	Chironomidae larvae	-0.14 (-0.29, 0.01)	-0.55 (-0.62, -0.49)	-0.52 (-0.87, -0.16)	0.22 (0.15, 0.33)	0.68 (0.55, 0.81)	-0.33 (-0.38, -0.29)	***38*.*46***	***0*.*0000***
PC2	Microcrustacean	-0.31 (-0.44, -0.18)	-0.50 (-0.55, -0.46)	1.94 (1.62, 2.26)	-0.50 (-0.55, -0.46)	***0*.*95*** ***(0*.*79*, *1*.*11)***	***-0*.*50*** ***(-0*.*55*, *-0*.*46)***	6.72	0.0347
PC2	Pelagic	***1*.*01*** ***(0*.*87*, *1*.*15)***	***0*.*52*** ***(0*.*46*, *0*.*59)***	-0.08 (-0.13, -0.04)	0.03 (0.02, 0.04)	0.28 (0.21, 0.35)	-0.18 (-0.20, -0.15)	***210*.*25***	***0*.*0000***
PC2	Benthic	0.16 (-0.01, 0.33)	-0.68 (-0.74, -0.62)	2.93 (2.50, 3.36)	-0.68 (-0.74, -0.62)	1.63 (1.40, 1.85)	-0.68 (-0.74, -0.62)	5.72	0.0572
PC2	δ^13^C	***-17*.*77*** ***(-18*.*40*, *-17*.*14)***	***1*.*64*** ***(1*.*38*, *1*.*94)***	-20.57 (-21.20, -19.94)	-0.94 (-1.25, -0.70)	-22.09 (-22.54, -21.64)	0.75 (0.62, 0.91)	***36*.*28***	***0*.*0000***
PC2	δ^15^N	***9*.*40*** ***(9*.*17*, *9*.*63)***	***0*.*55*** ***(0*.*50*, *0*.*62)***	***9*.*37*** ***(9*.*11*, *9*.*63)***	***0*.*55*** ***(0*.*50*, *0*.*62)***	***8*.*10*** ***(7*.*84*, *8*.*37)***	***0*.*55*** ***(0*.*50*, *0*.*62)***	6.05	0.0487
PC3	Chironomidae larvae	1.09 (0.96, 1.23)	-0.47 (-0.54, -0.42)	0.46 (0.32, 0.61)	-0.18 (-0.26, -0.12)	***0*.*77*** ***(0*.*66*, *0*.*89)***	***0*.*34*** ***(0*.*30*, *0*.*39)***	***28*.*22***	***0*.*0000***
PC3	Microcrustacean	-0.13 (-0.27, 0.01)	0.47 (0.43, 0.51)	-0.37 (-0.72, -0.02)	0.47 (0.43, 0.51)	1.01 (0.85, 1.18)	0.47 (0.43, 0.51)	11.92	0.0026
PC3	Pelagic	-0.16 (-0.29, -0.03)	0.45 (0.40, 0.51)	-0.02 (-0.04, 0.00)	0.02 (0.02, 0.03)	-0.18 (-0.25, -0.11)	-0.18 (-0.21, -0.16)	***178*.*04***	***0*.*0000***
PC3	Benthic	1.71 (1.54, 1.87)	-0.62 (-0.68, -0.57)	1.36 (1.03, 1.69)	-0.62 (-0.68, -0.57)	***-0*.*01*** ***(-0*.*28*, *0*.*26)***	***-0*.*62*** ***(-0*.*68*, *-0*.*57)***	11.29	0.0035
PC3	δ^13^C	-21.09 (-21.66, -20.52)	2.46 (2.05, 2.94)	-23.21 (-24.29, -22.13)	-1.11 (-1.47, -0.83)	-21.25 (-21.60, -20.90)	0.66 (0.54, 0.80)	***93*.*01***	***0*.*0000***
PC3	δ^15^N	8.84 (8.68, 9.00)	-0.65 (-0.73, -0.58)	7.34 (6.93, 7.75)	-0.65 (-0.73, -0.58)	9.69 (9.37, 10.01)	-0.65 (-0.73, -0.58)	3.65	0.1614

### Relationships between stable isotopes and other trophic markers

δ^13^C and δ^15^N were each significantly related to several diet and fatty acid components (Tables 3 and 5; Figs 1, 3 and 4). However, these relationships were not always consistent among species, especially when considering their relationship to the abundances of various fatty acids. When related to diet contents, δ^13^C exhibited positive relationships to chironomid larvae consumption (Fig 1a) and benthic consumption (Fig 1c), and a negative relationship to pelagic consumption (Table 3; Fig 1d). Although species-specific slopes and correlation strengths varied significantly in these relationships, their directions and magnitudes were similar across species. δ^15^N, conversely, exhibited no significant correlations with diet content in any species—although species-specific slopes differed for chironomid and pelagic consumption, no single species correlation was significant. Similarity in species-specific slopes also differed between δ^13^C and δ^15^N –all models for δ^13^C had significantly different slopes (Fig 1), whereas only relationships between δ^15^N and chironomid or pelagic consumption differed among species (Table 3).

Relationships between stable isotope ratios and single fatty acid profiles were variable (Table 5). DHA and δ^13^C values were significantly positively correlated in round goby, but non-significant and negatively correlated in spottail shiner and yellow perch, respectively; no other species-specific relationships were significant between δ^13^C and fatty acids (Fig 4a and 4c). Every model testing relationships between δ^13^C and fatty acids exhibited significant differences in slopes among species, with often contrasting directions between round goby and the other species (Table 5). In contrast, δ^15^N values were consistently negatively related to DHA, EPA, and ALA across species (Table 5). ALA and δ^15^N were significantly negatively correlated in both round goby and yellow perch (Fi. 3d), whereas only yellow perch exhibited a significant negative correlation between EPA and δ^15^N, and only round goby exhibited a significant negative correlation between DHA and δ^15^N (Fig 4b and 4d). No model testing relationships between δ^15^N and fatty acids found significantly different slopes among species.

### Relationships between fatty acids and diet items

Significant relationships existed between the relative abundance of ARA, EPA, and DHA and diet contents, whereas no significant relationships were observed between ALA and diet content (Table 4). EPA exhibited varied relationships to diet content among species. EPA was significantly positively correlated with chironomid consumption in round goby and to benthic consumption in both round goby and yellow perch (Fig 2a and 2e). However, EPA differed among species in its relationships to pelagic prey, exhibiting a significant negative correlation in round goby and non-significant, positive correlations in yellow perch and spottail shiner (Fig 2a, 2c, 2e and 2g). Similarly, microcrustacean consumption was significantly positively correlated with EPA in yellow perch, but these markers were non-significantly positively correlated in round goby. Lastly, DHA exhibited relatively consistent relationships among species (Fig 2b, 2d, 2f and 2h). DHA was negatively correlated to chironomid and benthic consumption in both yellow perch and round goby, and further negatively correlated to microcrustacean consumption in round goby only, although there was no evidence for different slopes in that relationship among species (Table 4). ARA was negatively correlated to the consumption of chironomids and microcrustaceans in both round goby and yellow perch (Fig 3a and 3c), and also negatively correlated to benthic consumption in round goby (Fig 3b). Again, although some significant differences in slopes were identified among species, the directions and magnitudes were generally similar.

The ratio of n-3:n-6 fatty acids was largely only related to diet contents in round goby, which exhibited significant positive correlations between n-3:n-6 and microcrustaceans and benthic consumption, and a negative correlation with pelagic consumption, but these correlations were not significant in the other species (Table 4; Fig 5).

### Fatty acid composition as a multivariate trophic marker

Separate PCAs were performed on fatty acid composition of fish in each subset of data (i.e., fish that had both fatty acid content and diet contents analyzed; fish that had both fatty acid and stable isotope ratios analyzed). The full suite of loadings for fatty acids on particular PCs are included as supplementary material (S2 Table). As mentioned in the Methods section, we retained the first three principal components (PCs) for each analysis and related them to other diet and stable isotope ratio values to examine whether a multivariate indicator of fatty acid composition can better discriminate and define the trophic niche and behavior of individual fish. There were several significant relationships between these multivariate fatty acid indicators and other trophic metrics, primarily in round goby and yellow perch, which are detailed below.

For the fatty acid-diet PCA, PC1 appeared to represent a gradient between fish high in C16 and C18 fatty acids such as 16:1n-7, 18:2n-6, and 18:3n-6, versus fish higher in C20 and C22 fatty acids like ARA, 22:5n-6, and 22:4n-6 (S2 Table). PC1 was significantly negatively correlated with higher proportions of chironomid larvae consumed in both round goby and yellow perch, and negatively correlated with microcrustacean and benthic consumption in round goby only, although the slopes of those relationships did not differ among species (Table 6; Fig 6). PC2 was most strongly negatively associated with EPA and 22:5n-3, and most strongly positively associated with several C20 acids, namely 20:4n-3, 20:3n-3, and 20:2n-6 (S2 Table). PC2 was negatively associated with microcrustacean consumption across all species, and significantly negatively correlated to microcrustacean consumption in yellow perch. In contrast, round goby exhibited a significant positive correlation between pelagic consumption and PC2, whereas the other species exhibited significantly different slopes and negative correlations (Table 6; Fig 7a and 7b). PC3 was strongly negatively associated with DHA and 16:0, and positively associated with multiple relatively low abundance acids, including 20:1, 15:0, and 18:1n-7. PC3 was significantly positively correlated with chironomid consumption in yellow perch, but negatively correlated to chironomids in spottail shiner and round goby (Fig 7c). PC3 was further significantly negatively correlated with benthic consumption in yellow perch, and this relationship was similar across species, although correlations were not significant in round goby or spottail shiner.

For the fatty acid-stable isotope PCA, PC1 was generally negatively associated with shorter chain fatty acids (C14 and C18) and positively associated with longer chain fatty acids (C20 or C22) (S2 Table). PC1 was significantly positively correlated to δ^13^C values in round goby, but not the other species, and showed no relationship to δ^15^N (Table 6; Fig 8a). DHA and 16:0 were most strongly positively associated with PC2 while a number of generally low-abundance fatty acids were negatively associated with PC2 (18:1n-7, 15:0, and 20:1 being the most strongly negatively associated; S2 Table). PC2 exhibited a species-specific interaction in relationship to δ^13^C, with a significant positive correlation in round goby but no correlation in other species (Table 6; Fig 8b). In contrast, PC2 was consistently and significantly positively correlated with δ^15^N across species—this was the only case where all three species exhibited significant correlations in the same direction (Fig 8c). PC3 was strongly negatively associated with 20:3n-6 and 20:4n-3, but not significantly correlated to either δ^13^C or δ^15^N (Table 6).

## Discussion

Understanding which trophic markers are consistently interrelated across species and systems can add value to ecological field studies on food web structure; however, whether such consistent interrelationships exist across ecosystems is unclear [46]. We have shown that, for three species of wild fishes inhabiting multiple, distinct habitats in a large lentic system, a large number of commonly used trophic metrics are indeed related to each other in expected directions. In particular, our findings support the long-held expectation that δ^13^C indicates reliance on benthic energy pathways [5,18,47]. δ^13^C was consistently positively related to chironomid and benthic items consumed and negatively related to pelagic items consumed for all three species examined in the current study. In addition, δ^15^N was generally not related to diet content but was related to several fatty acid indicators. We also observed several instances where relationships varied among species or opposed expected patterns.

We expected that tissue fatty acid content would be strongly related to diet composition in our samples. Freshwater fish lack the ability to produce certain fatty acids, notably C18 acids such as 18:2n-6, ALA, or 18:3n-6, and may acquire many long-chain polyunsaturated fatty acids, like ARA, DHA, and EPA, directly from their prey [26,48]. When assessing single fatty acids, we observed that EPA was positively related to microcrustacean and benthic consumption in round goby and yellow perch, whereas ARA and DHA were negatively related to microcrustaceans and benthic consumption across species. These relationships did not completely align with other studies (e.g., [31,33,34]), but this inconsistency may be explained by a single, important diet item in the fishes collected. Chydorids are benthic-oriented microcrustaceans that feed primarily on epiphyton [49,50]. Chydorids and other benthic invertebrates may serve as EPA-rich prey items to fish [31,33], and EPA is thought to be an important fatty acid for zooplankton growth and fitness [51]. ARA, in contrast, is generally considered to represent benthic pathways in aquatic systems [30,32] while DHA is thought to represent pelagic pathways; however, both ARA and DHA may be relatively low in microcrustaceans as compared to other available prey, meaning chydorids may offer relatively less of these compounds to predators despite their reliance on benthic energy sources [31]. Thus, our results suggest that, in this particular system, EPA in fish tissue may be reflective of reliance on chydorids, and a general reliance on chydorids as a benthic energy source may alter expected relationships between fatty acids and foraging behavior.

The lack of clear and consistent relationships between ALA and other trophic metrics in our study may partially reflect the metabolic importance of this compound in fish. ALA and 18:2n-6 can be used by freshwater fish to synthesize ARA, EPA, and DHA in a chain of reactions terminating in the elongation of DHA from EPA and other eicosanoids [29,48,52]. It is likely that fish internally regulate the abundance of these compounds in response to the specific composition of fatty acid contributed from their diet, allowing for adaptation to local environmental conditions [29]. This may complicate any relationship between various trophic metrics and concentrations of these fatty acids in the diets and tissues of consumers. This is important, as these and other polyunsaturated fatty acids have been used to examine a range of physio- and ecological processes in aquatic systems, from fish health, growth, and reproduction [53–55] to assessments of habitat quality [56]. We suggest that, while ALA undeniably represents an important fatty acid for fish development and fitness, inconsistent metabolism may make it an unreliable trophic marker in fishes, especially when taxonomic information or prey fatty acid profiles are unknown. In addition, though we do demonstrate relationships between DHA and some other metrics, we caution that, to mitigate confounding effects of metabolic processes, interpretations should rely on accurate assessment of DHA in prey.

Although we did not observe any significant relationships between diet content and δ^15^N, we did observe multiple correlations between δ^15^N and fatty acid indicators, particularly as negative correlations with ALA, EPA, DHA, and a positive association with PC axis (PC 2; representing a benthic to pelagic gradient), all of which would seem to suggest that trophic level may trend with energy source in this system. One possibility is that the observed correlation between δ^15^N and pelagic energy sources may be driven by food chain length, either via consumption of dreissenid mussels in round goby, or consumption of herbivorous zooplankton in round goby and spottail shiner. These pelagic prey sources feed primarily on phytoplankton and may incorporate a broad range of primary and secondary consumers (i.e., diatoms, rotifers, algae) compared to benthic organisms like chydorids, which feed primarily on epiphyton and represent a single step from primary producers to primary consumers [57]. The positive correlation between δ^15^N and pelagic resource use, as indicated by fatty acids, may therefore be driven primarily by consumption of diet items that are at odds with others in their respective categories. In addition, the pattern of relationships may be due to differences in the type and turnover rates of the trophic information these markers characterize. Fatty acid and stable isotope signatures turn over at slower rates than diets and reflect the outcome of potentially variable digestion and assimilation rates of different prey species [12], and therefore may be more likely to reflect energy sources utilized by a consumer than diet content as a function of their linkage via individual metabolic rates [25]. It should be noted, however, that δ^13^C was relatively well related to diet content, suggesting that a relationship between trophic level and fatty acid signatures of prey may be a more likely explanation.

Most previous studies have considered the trophic relationships between singular fatty acids and probable energy sources (reviewed in [9]). However, assessing variation in the entire fatty acid composition of individuals may provide a more comprehensive interpretation of their trophic behavior than assessment of individual fatty acids alone. We found several significant relationships between composite measures of fatty acid composition and other trophic markers, suggesting that a holistic approach to fatty acid composition may be appropriate. In the simplest case, the ratio of n-3:n-6 fatty acids was significantly negatively related to pelagic consumption and positively related to microcrustacean consumption. These patterns were expected, as benthic prey are generally high in n-3 fatty acids compared to herbivorous zooplankters [31,33,35]. The relatively high number of significant relationships we observed between the multivariate fatty acid principal components and various diet content categories also suggests a more holistic approach to fatty acids has merit in food web studies, especially when additional examination of taxonomic, spatial and temporal variability in fatty acid signatures is possible [46]. These relationships also appear to make ecological sense. For example, the fatty acid-diet PC1, which could be interpreted to represent a benthic (negative loadings of 16:1n-7; [33]) to pelagic (positive loadings of C22 fatty acids; [31]) gradient, was generally negatively related to chironomid and benthic consumption. PC2, which represented a similar benthic invertebrate (negative loadings of EPA, DPA, and 18:1n-7; [9]) to pelagic (positive loadings of C20 eicosanoids; [31]) gradient was positively related to pelagic consumption. And, in the only instance where all three species exhibited consistent, significant correlations in the same direction, the fatty acid-stable isotope PC2, which represented a benthic (negative loadings of 18:1n-7) to pelagic (positive loadings of DHA) gradient was positively related to δ^15^N. Our results reinforce the suggestion that using a suite of fatty acids improves understanding of an organism’s trophic position over considering a single fatty acid indicator [45,58,59].

Even though we only assessed three species, our results underline the importance of accounting for inter-taxa variability when interpreting trophic markers [34,36]. Opposing directions of associations when comparing species, or correlations in only one species but not others occurred primarily under two scenarios—when pelagic prey or microcrustacean consumption was considered as a response, and when round goby differed from yellow perch in slope direction. Specifically, the relationships between EPA, DHA, fatty acid PCs, δ^15^N, and indicators of pelagic or benthic consumption were in different directions between round goby and either or both of the other two species. While the goal of this paper is not to specifically understand mechanisms by which relationships between different trophic metrics came about, we hypothesize that these particular interactions are the result of a combination of taxonomic differences in fatty acid metabolism between fish species, and unique prey item life history. First, invasive dreissenid mussels are highly efficient at filtering the water column and have come to dominate Great Lakes benthic systems [57], likely representing a unique fatty acid signature that complicates general understanding of patterns in this system. Dreissenids are at once rich in fatty acids associated with both benthic (ARA) and pelagic (ALA, DHA, and other long-chain C22 molecules) energy pathways [31]. Round goby were the only of the three fishes examined in our study to consume many dreissenids, complicating our ability to generalize our “pelagic” fatty acid results across species. Second, as previously mentioned, freshwater fish can synthesize long-chain C20 and C22 fatty acids from C18 precursors. Marine fish, due to adaptations to fatty acid availability in marine environments, generally lack this ability, instead acquiring these essential fatty acids from their diet [29,48,60]. In their native range in the Black and Caspian Seas, round goby are primarily a euryhaline demersal species, although they have invaded freshwater habitats across Europe and the Laurentian Great Lakes [61]. If they lack the appropriate elongase activity to synthesize long chain fatty acids compared to the native freshwater species included in this study due to their history as a marine or brackish-water species, this could manifest as differences in relationships between round goby fatty acids and other trophic biomarkers compared to yellow perch and spottail shiner. To our knowledge, the activity of various elongases has not been quantified in round goby, although there is some evidence that they have limited capacity to synthesize DHA or ARA, supporting our hypothesis [62]. Further investigation, including consideration of phylogenetic relationships between fish species as a potential mechanism for how fatty acids are synthesized, is likely warranted.

By examining relationships among diet contents, stable isotopes, and fatty acid composition within individual fish across three species inhabiting a large lake, we were able to quantify sometimes complex relationships between trophic markers that are commonly used to assess aquatic food web patterns. As previously mentioned, we purposely did not include site and season as analyzing factors in the current study, as our intent was to understand associations between metrics without any external understanding of what was shaping relationships. However, in an unpublished analysis where we did include site and season of collection as explanatory factors, the correlations observed were generally unchanged with what is presented herein. In summary, while diets and stable isotopes were generally well-related and the directions of correlations were as expected, the relationships between diet contents or stable isotope ratios and fatty acid signatures of individuals were not so clear. While fatty acid analysis is gaining popularity as another metric to assess food web structure [58], our study illustrates that precise inferences about trophic relationships using fatty acids may require extra information. Specifically, as noted in previous work [12], understanding the effects of taxonomic variation in prey and predator signatures, and elucidating metabolic and synthesis pathways among species with unique natural histories, especially when these species are introduced outside their native range, could significantly improve the usefulness of fatty acids in food web studies.

## Supporting information

S1 Table Fish sample sizes and lengths. Sample sizes and mean total lengths (± 1 S.D.) of Lake Michigan fish whose trophic markers were directly compared, grouped by collection location (site) and species (ROG = round goby, STS = spottail shiner, YEP = yellow perch).
(PDF)

S2 Table Fatty acid principal components analysis. Principal component loadings for fatty acids in the Diet-Fatty acid and Stable isotope-Fatty acid data sets. Values in bold italics are greater than |0.2|.
(PDF)
