# Supplementary material for: Species identity matters when interpreting trophic markers in aquatic food webs
Source: PLoS One. 2018 Oct 5;13(10):e0204767. doi: 10.1371/journal.pone.0204767 (PMC6173397; doi:10.1371/journal.pone.0204767)
Supplement: S1 Table — Sample sizes and mean total lengths (± 1 S.D.) of Lake Michigan fish whose trophic markers were directly compared, grouped by collection location (site) and species (ROG = round goby, STS = spottail shiner, YEP = yellow perch). (PDF) [file pone.0204767.s001.pdf]

Feiner et al. Species identity matters when interpreting trophic markers in aquatic food webs

S1 Table. Sample sizes and mean total lengths (± 1 S.D.) of Lake Michigan fish whose trophic markers were directly compared, grouped by collection location (site) and species (ROG = round goby, STS = spottail shiner, YEP = yellow perch).

		Sample sizes			Total lengths (mm)		
	Site	ROG	STS	YEP	ROG	STS	YEP
Diets-Fatty acids	AR	24	0	17	78.5 ± 20.7	--	89.4 ± 16.7
	IL	75	4	63	78.9 ± 24.3	107.6 ± 8.9	100.8 ± 29.7
	IN	62	14	84	70.8 ± 14.2	94.7 ± 17.7	93.3 ± 39.2
	MI	65	4	29	73.4 ± 23.2	89.8 ± 8.7	97.1 ± 48.6
	SB	16	0	0	68.6 ± 7.5	--	--
	WI	43	4	30	75.8 ± 23.6	93.3 ± 2.5	247.6 ± 68.2
Diets – Stable isotopes	AR	11	0	8	88.3 ± 19	--	91.7 ± 23
	IL	27	4	26	77.1 ± 21.3	107.6 ± 8.9	96.3 ± 27.4
	IN	50	13	48	75.7 ± 17.6	97.4 ± 15.2	106.1 ± 51.5
	MI	29	4	21	78.9 ± 25.8	89.8 ± 8.7	103.7 ± 55.9
	SB	7	0	0	62.4 ± 6.9	--	--
	WI	43	4	16	72.3 ± 26.2	93.3 ± 2.5	232.9 ± 83.9
Stable isotopes – fatty acids	AR	9	0	13	94.8 ± 20.9	--	89.7 ± 18.7
	IL	25	8	23	76.4 ± 22.6	107.2 ± 12.5	99.5 ± 23.5
	IN	43	23	41	75.7 ± 14.9	100 ± 17.6	114.6 ± 52
	MI	26	11	24	86.3 ± 29.1	91.7 ± 12.5	129.2 ± 64.3
	SB	11	0	0	77.1 ± 31.3	--	--
	WI	28	8	19	78.3 ± 27.2	97.1 ± 9	218.1 ± 93.1
