# Supplementary material for: Species identity matters when interpreting trophic markers in aquatic food webs
Source: PLoS One. 2018 Oct 5;13(10):e0204767. doi: 10.1371/journal.pone.0204767 (PMC6173397; doi:10.1371/journal.pone.0204767)
Supplement: S2 Table — Principal component loadings for fatty acids in the Diet-Fatty acid and Stable isotope-Fatty acid data sets. Values in bold italics are greater than |0.2|. (PDF) [file pone.0204767.s002.pdf]

Feiner et al. Species identity matters when interpreting trophic markers in aquatic food webs

S2 Table. Principal component loadings for fatty acids in the Diet-Fatty acid and Stable isotope-Fatty acid data sets. Values in bold italics are greater than |0.2|.

	Fatty acid-Diet			Fatty acid-stable isotope		
Fatty acid	PC1	PC2	PC3	PC1	PC2	PC3
12:0	0.021	-0.057	0.155	0.040	***-0.203***	0.041
14:0	-0.184	***0.284***	0.138	***-0.254***	-0.130	***0.234***
15:0	0.163	0.004	***0.286***	0.121	***-0.314***	-0.005
16:0	-0.139	0.107	***-0.339***	-0.132	***0.287***	0.125
16:1n-7	***-0.325***	0.026	0.100	***-0.218***	-0.071	***0.310***
16:1n-9	0.031	***0.250***	-0.187	-0.106	0.178	-0.035
17:0	***0.242***	-0.003	0.176	0.087	***-0.237***	***-0.281***
17:1	-0.036	0.089	***0.252***	-0.140	***-0.259***	-0.142
18:0	0.183	***-0.213***	-0.141	0.156	0.087	***-0.285***
18:1n-7	-0.131	***-0.240***	***0.265***	-0.047	***-0.324***	-0.070
18:1n-9	-0.091	***0.268***	0.075	***-0.259***	-0.072	-0.139
18:2n-6	***-0.259***	0.096	0.142	***-0.261***	-0.151	0.042
18:3n-3	-0.174	0.116	0.187	-0.128	-0.189	0.177
18:3n-6	***-0.208***	-0.060	0.171	0.007	-0.145	***0.301***
18:4n-3	-0.018	0.097	***0.212***	0.065	-0.145	***0.288***
20:1	***0.228***	0.048	***0.320***	0.107	***-0.288***	-0.164
20:2n-6	0.178	***0.293***	0.071	-0.156	0.011	***-0.319***
20:3n-3	0.076	***0.352***	0.108	***-0.230***	-0.067	***-0.275***
20:3n-6	0.179	***0.205***	***0.215***	-0.124	-0.168	***-0.348***
20:4n-3	0.047	***0.360***	0.082	***-0.244***	-0.058	-0.177
20:4n-6	***0.343***	-0.072	-0.087	***0.317***	0.052	-0.162
20:5n-3	-0.083	***-0.349***	0.138	0.165	***-0.226***	0.101
22:1n-11	0.175	-0.067	0.101	0.169	-0.007	-0.011
22:1n-9	0.197	-0.080	0.196	***0.250***	-0.144	0.104
22:4n-6	***0.290***	0.008	0.095	***0.276***	-0.067	-0.015
22:5n-3	0.079	***-0.284***	0.143	***0.256***	-0.174	-0.022
22:5n-6	***0.318***	0.026	-0.027	***0.323***	0.036	0.027
22:6n-3	***0.209***	0.139	***-0.344***	0.076	***0.392***	-0.121
SD	2.388	2.139	2.043	2.489	2.192	1.994
Prop. variance	0.204	0.163	0.149	0.221	0.172	0.142
